# Prevalence, risk factors and spatial analysis of liver fluke infections in Danish cattle herds

**DOI:** 10.1186/s13071-015-0773-x

**Published:** 2015-03-15

**Authors:** Abbey Olsen, Klaas Frankena, Rene’ Bødker, Nils Toft, Stig M Thamsborg, Heidi L Enemark, Tariq Halasa

**Affiliations:** Quantitative Veterinary Epidemiology group, Wageningen University, Wageningen, The Netherlands; Section for Epidemiology, National Veterinary Institute, Technical University of Denmark, Frederiksberg C, Denmark; Veterinary Parasitology Research Group, Department of Veterinary Disease Biology, University of Copenhagen, Frederiksberg C, Denmark; Section for Bacteriology, Pathology and Parasitology, National Veterinary Institute, Technical University of Denmark, Frederiksberg C, Denmark

**Keywords:** Fasciola hepatica, Cattle, Denmark, Prevalence, Risk factors, Spatial model

## Abstract

**Background:**

*Fasciola hepatica*, a trematode parasite (liver fluke), infects a wide range of host species causing fasciolosis. The disease is prevalent world-wide and causes considerable economic losses to the livestock industry. Fasciolosis is regarded as an emerging food-borne zoonosis. To promote awareness among farmers and to implement strategies to control the infection, this study examined the prevalence, spatial distribution and risk factors for *F. hepatica* infection in Danish cattle herds.

**Methods:**

A retrospective population based study was performed using meat inspection data of approximately 1.5 million cattle slaughtered in the period 2011 to 2013. Annual cumulative prevalence of recorded liver fluke findings was calculated for each year. Global and local spatial cluster analysis was used to identify and map spatial patterns of *F. hepatica* positive and negative herds to explore environmental indicators of infection. Herd level, trade and environmental risk factors were evaluated for association with infection using logistic regression. Herd infection status as predicted from the final risk factor model was compared with the observed status using heat maps to assess how well the model fitted the observed spatial pattern.

**Results:**

During the investigated period (2011–2013), an increase in annual herd prevalence was noted (2011–25.6%; 2012–28.4%; 2013–29.3%). The spatial analysis suggested significant clustering of positive and negative herds. Presence of streams, wetlands and pastures on farms showed a significant association with the presence of infection in cattle herds. Buying animals from positive herds was a risk factor on conventional farms. Additionally, risk of being infected with *F. hepatica* was higher in non-dairy herds of medium size (≥30 and < 100) when compared to dairy and large (≥100) cattle herds. The observed spatial pattern could be reproduced by predictions of the risk factor model.

**Conclusions:**

This study showed an increase in annual herd level prevalence (2011 to 2013) indicating that an increasing proportion of herds are infected with *F. hepatica* infection every year in Denmark. Fasciolosis was found to be associated with both herd and environmental factors where the infection was influenced by local factors that clustered geographically.

## Background

Liver fluke infection, also known as fasciolosis or distomatosis, is a world-wide prevalent parasitic disease infecting a wide range of host species, and is regarded as an emerging food-borne zoonosis [1,2]. Over 17 million people are affected globally, where humans become accidental hosts by ingestion of contaminated water, aquatic vegetation or occasionally through consumption of raw or undercooked liver products [3]. The geographical distribution of *F. hepatica* is strongly linked to climate and environmental conditions such as presence of water bodies, pastures and wetlands. These conditions create a favourable environment for the development and transmission of free living fluke stages and for the growth and reproduction of the intermediate host snail (*Galba truncatula*) [4,5]. Apart from climate and environmental factors, animal level factors like age and breed and herd level factors such as stocking rate and type of farming system are also associated with occurrence of the infection [6,7].

In cattle, fasciolosis results in chronic infection which is most often sub-clinical, and therefore animals are often left untreated [1,8]. The disease causes considerable economic losses to the livestock industry, because of reduced productivity, liver condemnation and reduced carcass value [1,9]. In Switzerland, the financial loss per infected cow was estimated to be up to 376 euros per annum [10].

In Denmark, during the period 2000–2003, the prevalence of bovine fasciolosis at herd and animal level was estimated to be 12%–24% and 1.7%–4.3%, respectively where the infection was positively associated with grazing, wet-lands and soil composition of the geographical region ([11], unpublished data). Despite the substantial economic and animal welfare effects of the disease, up to date knowledge on its prevalence and risk factors related to its occurrence and distribution in Denmark are scarce. Therefore, the objectives of this study were to estimate the prevalence of fasciolosis in the Danish cattle population and to identify and quantify potential risk factors at herd level by evaluation of meat inspection data. Meat inspection serves as an important disease detection tool because it has high test specificity (SP = 100%) for liver flukes [12]. However, the sensitivity in individual cattle is low (SE = 60%) and is strongly influenced by the quality of the meat inspection which is shown to vary significantly between the abattoirs [12,13]. Therefore, aggregation of individual cattle meat inspection data to herd level improves herd level sensitivity. Hence, considering the less than perfect test sensitivity characteristic of meat inspection and the infectious nature of the disease, the present study was conducted at herd level to contribute towards control of the disease; in an effort to improve cattle herd health, performance and welfare and also prevent human liver fluke infection.

## Methods

### Study design and data collection

**Figure 1:**
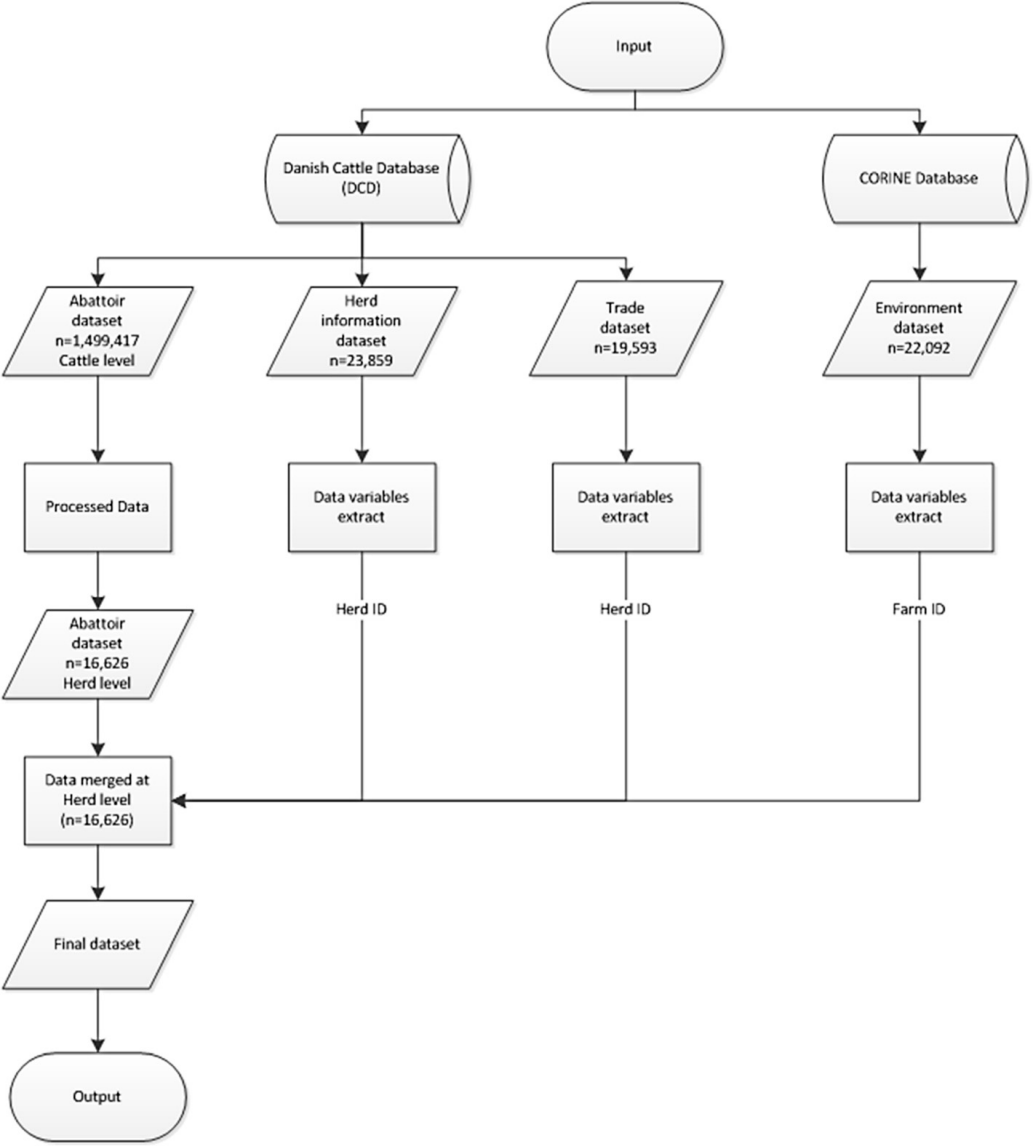
**Diagram showing how the final dataset was created through extraction of variables and merging of data from the two master databases, the Danish Cattle Database and the CORINE vector database.**

The study included all Danish herds with at least one bovine slaughtered in the years 2011–2013. Cattle and environmental data were extracted from the Danish cattle database (DCD) and the CORINE database, respectively. An overview of the variables contained in each dataset is presented in Table 1. Figure 1 illustrates the datasets used in the study and how they were merged to form one final dataset that was used for the analysis.

**Table 1:** **Information on the datasets and the variables considered for creating the final dataset for a study on** ***Fasciola hepatica*** **infection in Danish cattle herds**

**Abattoir dataset**	**Herd information dataset**	**Environment dataset**	**Trade dataset**
**(n = 1,499,417 cattle)**	**(n = 23,859 herds)**	**(n = 22,092 farms)**	**(n = 19,593 herds)**
*Farm characteristics:* Farm identification number; Farm coordinates	*Farm characteristics:* Farm identification number	*Farm characteristics:* Farm identification number; Farm coordinates	*Trade characteristics:* Purchase of an animal from another herd (0 = No, 1 = Yes); Purchase of an animal from an infected herd (0 = No; 1 = Yes; NA = Unknown^4^)
*Animal characteristics*: Animal identification number; Sex; Date of birth; Date of Slaughter; *F. hepatica* status of the animal at slaughter	*Count day:* Herd size recording date (beginning of every calendar month from 2005 to 2013)	*Environmental variables* ^3^ (0 = Absent, 1 = Present): Streams, Wetlands; Lakeshore; Cropland; Pastures; Grasslands; Forests; Artificial surface; Recreation land; Grass; Farmland; Forest; Dry-land; Fresh-water meadows; Ocean	*Animal characteristics: F. hepatica* status of the animal at slaughter (0 = Negative, 1 = Positive)
*Herd Characteristics:* Herd identification number; Farm type (Organic, Conventional); Production type: (Dairy, Non-dairy herds^1^)	*Herd characteristics:* Total number of bulls < 6 months of age; Total number of bulls > 6 months of age; Total number of heifers < 6 months of age; Total number of heifers > 6 months; Total number of cows; mean and median herd size^2^		*Herd Characteristics:* Herd identification number; Median herd size

^1^Non-dairy herds = Includes large and small beef herds, heifer raising herds, large and small veal production herds.

^2^Herd size = Total number of bulls below 6 months of age + number of bulls above 6 months of age + total number of heifers below 6 months of age + total number of heifers above 6 months + total number of cows.

^3^Environmental variables = Located within 500 square meters around each farm; *source*: CORINE database (2000).

^4^Unknown = Status of the herd from which the animal was purchased is not known and or included herds that did not trade with other herds.

The abattoir dataset obtained from the DCD was used to extract register data of all cattle slaughtered in Denmark. For this study a bovine was deemed positive for fasciolosis when at meat inspection the liver was condemned and recorded as infected due to typical lesions (enlarged fibrotic bile ducts and cholangiohepatitis) and/or when one or more flukes in the liver were detected, otherwise it was considered negative. Other inspection codes for acute and chronic hepatitis and liver abscess were considered non-specific markers for fasciolosis and therefore were not included in the disease status classification. Additionally, all bovines in Denmark have a unique identification and registration number and using that number it is possible to identify in which herd each bovine is located at any time. Cattle must be tagged with two ear tags no later than 20 days after birth and before they leave the holding of origin. One of the ear tags must be electronic. At slaughter, this unique number is recorded automatically without any interference of the veterinary inspector, and thus the herd from which the bovine was sent is identified. Moreover, the geographical coordinates (X and Y co-ordinates) of the farm were available in the database. The 19,593 herds in the abattoir dataset represented 82% of all herds which were registered active during 2011 to 2013.

The herd information dataset was sourced from the DCD and included data on herd composition (number of animals per age group). Herd size categories (Small ≤ 30 animals, Medium > 30 and < 100 animals, Large ≥ 100 animals) were determined by calculating the median from the total number of animals present in the herd throughout the study period (counted each first day of the month).

The trade dataset was obtained from the DCD and included information about movements of animals between the herds and the abattoirs. This dataset was merged with the abattoir dataset to create a variable to indicate whether or not a farm had purchased an animal from an infected herd (one or more infected cows).

The environment dataset was obtained from the CORINE land cover vector database which consists of environmental data from the European landscape. The classification of data into classes of the CORINE land cover nomenclature is done through photo-interpretation of satellite images on a computer, with additional ancillary data. The images are interpreted based on transparencies overlaid on hardcopy prints of satellite images; whereas, the ancillary data helps to identify and confirm the contents of certain land cover features, detected on the images [14]. The environment data included quantitative data on land cover within a zone of 500 meters around each farm (n = 22,092 farms) registered in the year 2000. All the environmental variables were categorized into binary variables, based on presence or absence in the 500 meter zone (Table 1). The environmental variables included in the study are described in the CORINE land cover technical guide [14]. In total 2,950 herds present in the abattoir dataset were missing in the environment dataset.

The final dataset was created by merging all the datasets to include information on 16,626 herds (out of 19,593) that were used for statistical analysis. In total 2,967 records were excluded due to missing information on environmental parameters, herd size or geographical coordinates. For herd level analysis, abattoirs (n = 79) were categorized (A-H, Other) based on number of animals slaughtered. Abattoir categories A-H were individual abattoirs whereas “Other” included 70 small slaughterhouses processing less than 7,500 animals over the 3 year study period (i.e. less than 10 per day). To each herd record, the abattoir category where most animals had been brought to was added as ‘preferred abattoir’. However, for herds that had brought equal numbers of animals to two or more abattoirs, the most recently used abattoir was chosen as the preferred one. The final herd level dataset included information about farm and herd identification numbers, environmental variables, trade information, abattoir information, infection status, herd size, farm-type, production type, and location of the herds (X and Y coordinates).

### Statistical analysis

#### Proportion of positives

The proportion of apparent positives per annum was determined both at animal and herd level. For the spatial analyses and risk factor studies, a cattle herd was classified as positive when a minimum of one animal from the herd tested positive for *F. hepatica* at meat inspection during the study period, otherwise the herd was considered negative.

#### Spatial analysis

*Fasciola hepatica* infection is driven by environmental factors [11,15] and environmental variables show clustering across geographical areas [16]. Spatial analysis was used to explore whether *F. hepatica* infection was clustered in space as this could help identify environmental factors associated with the infection. Both global and local spatial autocorrelation techniques were used to detect infected and non-infected herd clusters [16]. Global clustering statistics detect spatial clustering that occurs anywhere in the study area but cannot identify where the clusters occur [17]. Hence, local mapping techniques were used to identify and map potential clustering to an area on a map.

Global spatial autocorrelation (clustering) of *F. hepatica* positive herds was quantified using two complementary spatial statistical methods; the global Moran’s I and general G statistic in ArcGIS 10.1 Spatial Analyst software (n = 16,626 herds) [16]. For both methods, an inverse squared Euclidean distance [(1/ (Distance)^2^] with a threshold value of 5000 meters between two neighbouring herds was selected [18].

The global Moran’s I was interpreted by an index: values close to +1.0 indicate clustering and values near-1.0 indicate dispersion. The Z-score and P-value were used to evaluate the significance of Moran’s Index. The general G statistic was interpreted relative to its expected value where G larger than the expected value suggested clustering of positive herds and G smaller than the expected value indicated clustering of negative herds. Z test statistic was used for significance testing [18].

SatScan 9.3 was used for the detection and mapping of statistically significant (95% level) local clusters [19]. A purely spatial scan statistics with the Bernoulli model was used to simultaneously scan for both high and low rate clusters (positive herds, n = 6,835 and negative herds, n = 9,791). The SatScan output was run to detect circular clusters on the map by selecting the circular spatial window without overlapping clusters and maximum radius of 50% of population at risk in the settings window [19]. Statistical significance was explored by 999 Monte Carlo replications.

From the SatScan output, relative risk (RR) and P-values were extracted and each farm was categorized as being in a hot spot (RR ≥ 1.00, P ≤ 0.05) a cold spot (RR < 1.00, P ≤ 0.05) or neither and mapped in ArcGIS software (version 10.1).

#### Risk factor analysis

A risk factor analysis at herd level was performed for the three-year period using logistic regression in SAS (version 9.2). The outcome variable was a binary variable reflecting *F. hepatica* herd status (0 = Negative, 1 = Positive). The predictor variables screened for association with *F. hepatica* infection were the herd level, trade and environmental factors (Table 1). To account for the observed spatial autocorrelation the distance to the nearest positive neighbour for each herd was calculated. This variable was deduced by using herd identification number, infection status and geographical coordinates of the herds. This continuous variable was used to adjust for spatial autocorrelation in the herd infection status and was added to the model as a fixed effect. Herd and environmental categorical variables were first tested for all possible pairwise correlations. If the correlation coefficient (Cramér’s V) was larger than 0.50, then the variable having higher biological precedence over the other variable was selected for further modelling. All the selected variables were then fitted into a multivariable model. A backward-elimination procedure was used to simplify the initial model. Non-significant variables (P > 0.05, based on Wald Type III Chi square) were deleted sequentially, beginning with the variable showing the largest P-value. Variables were removed permanently from the model if they were not confounders; where confounding was defined as a change in any remaining parameter, estimated greater than 20% when compared to the previous model. This process of deleting, refitting and verifying was repeated until all variables in the model were either significant (P < 0.05) or deemed a confounder. Next, biological plausible interaction terms were added and retained when they were significant (P < 0.05).

#### Evaluation of spatial predictions

Herd infection status as predicted from the final risk factor model was visually compared with the observed status using a heat map. This was done to assess whether the model was able to reproduce the observed spatial pattern and therefore able to capture the key environmental parameters involved in the transmission of the disease. Hence, observed status (positive and negative herds) and predicted infection probability for each herd (0 to 100%) was interpolated with the inverse distance weighted technique [IDW, (1/ (Distance)] in ArcGIS software (version 10.1) using a maximum distance of 10,000 meters to include all herds situated within this radius.

## Results

### Distribution of *F. hepatica* infection

An overview of herd level descriptive statistics is shown in Table 2. During the investigated period, there was an increase in per annum prevalence estimates for fasciolosis at both animal and herd level (P < 0.001, based on Wald Chi square from a logistic regression model). At animal level the per annum prevalence (P) estimates during 2011 to 2013 were 3.2% (P = 16,300/516,461; 95% CI = 3.1%–3.2%), 3.9% (P = 19,139/492,184; 95% CI = 3.8%–3.9%) and 3.9% (P = 19,326/490,772; 95% CI = 3.9%–4.0%), respectively. Whereas, at herd level the per annum prevalence estimates during 2011 to 2013 were 25.6% (P = 4,271/16,683; 95% CI = 24.9%- 26.3%), 28.4% (P = 4,506/15,867; 95% CI = 27.7%–29.1%) and 29.3% (P = 4,492/15,331; 95% CI = 28.6%–30.0%), respectively.

**Table 2:** **Descriptive characteristics of herds (n = 16,626) and percentage tested positive for** ***F. hepatica*** **infection, as determined at meat inspection (years 2011–2013) in Danish abattoirs**

		**Farm-type**		**Production-type**	
**Variable**	**Category**	**Organic**	**Conventional**	**Dairy**	**Non-dairy** ^**1**^
		**n (% infected)**	**n (% infected)**	**n (% infected)**	**n (% infected)**
Trade	No	215 (34.9)	5,321 (27.7)	331 (47.1)	5,205 (26.7)
	Yes	652 (59.7)	10,438 (46.9)	3,971 (58.9)	7,119 (41.4)
Pastures	Absent	810 (52.6)	14,832 (39.5)	4,012 (57.1)	11,630 (34.4)
	Present	57 (66.7)	927 (54.6)	290 (69.7)	694 (49.3)
Wetlands	Absent	504 (51.6)	9,555 (37.7)	2,696 (55.2)	7,363 (32.2)
	Present	363 (56.2)	6,204 (44.7)	1,606 (62.7)	4,961 (39.7)
Streams	Absent	580 (51.7)	11,277 (39.0)	3,030 (56.9)	8,827 (33.7)
	Present	287 (57.1)	4,482 (44.1)	1,272 (60.5)	3,497 (39.2)
Dry-land	Absent	323 (53.6)	5,741 (38.6)	1,701 (57.9)	4,363 (32.3)
	Present	544 (53.5)	10,018 (41.5)	2,601 (58.1)	7,961 (36.9)
Crop-land	Absent	4 (50.0)	40 (35.0)	1 (100.0)	43 (34.9)
	Present	863 (53.5)	15,719 (40.4)	4,301 (58.00)	12,281 (35.2)

^1^Includes large and small beef herds, heifer raising herds, large and small veal production herds.

### Spatial cluster analysis

#### Global clustering

A significant positive identified Moran’s I value identified positive spatial autocorrelation for *F. hepatica* (Moran’s I = 0.12; Z = 4.39; P < 0.05). Additionally, the general G results revealed higher levels of clustering for infected herds than for the non-infected herds (General G = 0.00001; Z = 5.43; P < 0.05). The positive global clustering outcome of both the methods suggested spatial autocorrelation of key risk factors.

#### Local clustering

**Figure 2:**
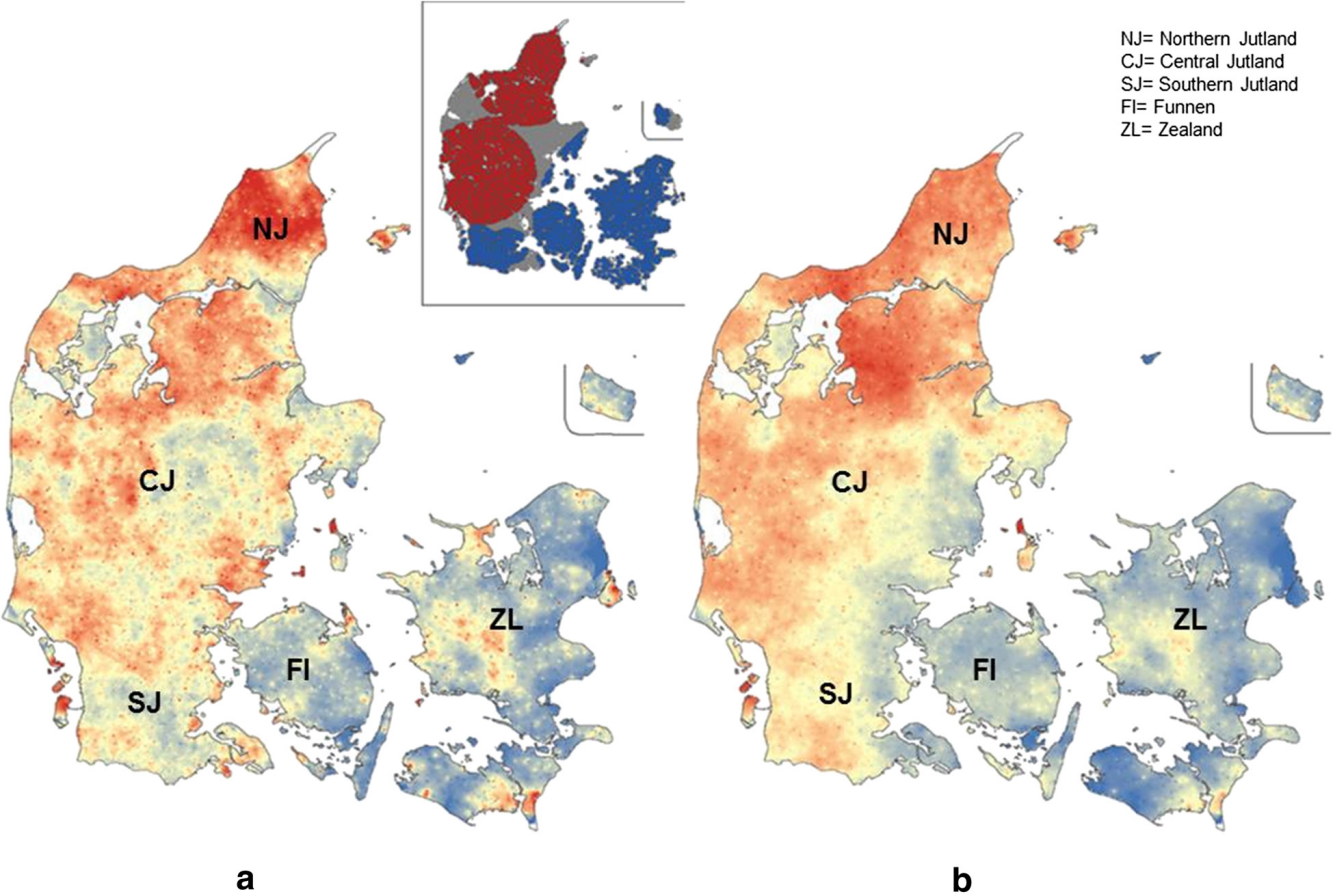
**Heat maps of observed (a) and predicted (b) status of** ***Fasciola hepatica*** **infection in Danish cattle herds (n = 16,626) where areas coloured in red and blue indicate hot (RR ≥ 1.0) and cold spots (RR < 1.0), respectively.** The insert in Figure 2 a shows a SatScan map of significant spatial local clustering of infected (red) and non-infected (blue) herds.

As spatial autocorrelation was recognized, local clusters were identified and mapped. The results from the circular scan showed that 6,126 herds were situated in hot spots where the RR for *F. hepatica* infection was 1.4; whereas 1,055 herds were situated in cold spots where the RR was 0.6. The plotting of hot spots on a map of Denmark revealed a strong overall spatial trend with concentration of high RR for *F. hepatica* infection around the North and Central Jutland region of Denmark (Figure 2 a, insert). Whereas, plotting of cold spots revealed low RR for *F. hepatica* infection in the Southern Jutland, Funen, Islands and the Zealand region.

### Risk factors for *F. hepatica* infection in Denmark

Eleven variables and three interaction terms were significantly (P < 0.05) associated with *F. hepatica* status and therefore remained in the final model (Table 3). The Hosmer-Lemeshow statistic for logistic regression showed that the model fitted the data poorly (P-value = 0.02) suggesting that spatial autocorrelation was not fully addressed with the current model.

**Table 3:** **Herd and environmental factors associated with the presence of** ***F. hepatica*** **infection in Danish bovine herds (n = 16,626) as diagnosed by meat inspection (2011 to 2013), in the final logistic regression model**

**Variable**	**Class**	**Frequency** ^**1**^	**Percentage positive**	**Coefficient (S.E)**
Intercept				−0.95 (0.41)^b^
Farm-type	Conventional	15,759	40.43	−0.18 (0.14)
	Organic	867	53.52	ref
Production-type	Non-dairy^2^	12,324	35.22	0.21 (0.14)
	Dairy	4,302	58	reference
Distance to the nearest fluke positive herd^3^				−0.19 (0.02)^a^
Slaughterhouse	A	1,862	42.16	0.58 (0.07)^a^
	B	2,669	56.13	1.21 (0.06)^a^
	C	2,006	47.51	0.66 (0.07)^a^
	D	2,378	40.92	0.44 (0.07)^a^
	E	725	50.07	0.91 (0.09)^a^
	F	1,025	41.76	1.03 (0.08)^a^
	G	575	40.17	0.62 (0.10)^a^
	H	1,460	62.12	1.53 (0.07)^a^
	Other	3,926	17.75	reference
Streams	Present	4,769	44.87	0.16 (0.04)^a^
	Absent	11,857	39.6	reference
Wetlands	Present	6,567	45.29	0.31 (0.04)^a^
	Absent	10,059	38.38	reference
Cropland	Present	16,582	41.12	−0.88 (0.36)^b^
	Absent	44	36.36	ref
Pastures	Present	984	55.28	0.38 (0.07)^a^
	Absent	15,642	40.22	reference
Dry-land	Present	10,562	42.08	−0.08 (0.04)^b^
	Absent	6,064	39.43	reference
Herd-size	Small (<30)	9,028	26.09	reference
	Medium (≥30 to < 100)	3,200	53.41	1.17 (0.27)^b^
	Large (≥100)	4,398	63.01	1.92 (0.23)^b^
BuyInfYN^4^	Yes	1,659	61.18	0.68 (0.25)^b^
	Unknown	3,106	55.02	−0.15 (0.21)^b^
	No	11,861	34.66	reference
Farm-type*BuyInfYN	Conventional*Yes	1,547	60.31	0.09 (0.26)
	Conventional*Unknown	2,962	54.90	0.65 (0.22)^a^
	Conventional*No	11,250	33.88	reference
Farm-type*Herd-size	Conventional*Small	8,726	26,08	reference
	Conventional*Medium	3,045	52.97	−0.56 (0.23)^b^
	Conventional*Large	3,988	62.24	−0.48 (0.19)^a^
Production-type*Herd-size	Non-dairy*Small	8,760	26.03	reference
	Non-dairy*Medium	2,640	55.72	0.42 (0.17)^a^
	Non-dairy*Large	924	63.85	−0.06 (0.17)

^1^Number of herds per category.

^2^Includes large and small beef herds, heifer raising herds, large and small veal production herds.

^3^Distance in km, mean = 1.28, median = 1.05, 5th percentile = 0.26, 95th percentile = 3.06 km.

^4^Trade with infected herds (Yes, No, Unknown) where Unknown represented herds with unknown infection status.

^a,b^P-value ≤ 0.01 and ≤ 0.05, respectively.

Out of 14 environmental variables, five variables (cropland, dry-land, streams, wetlands and pastures) showed a significant association with *F. hepatica* status (P < 0.05). Presence of streams, wetlands and pastures on a farm was a risk factor for a positive *F. hepatica* status of herds. However, the presence of cropland and dry-land on a farm showed a negative association with *F. hepatica* status. The estimate for distance to the nearest positive neighbour indicated that the probability of slaughtering a positive animal was reduced as the distance to the nearest positive neighbour increased. Additionally, a significant association was present between *F. hepatica* status and abattoir, where the percentage of positives varied between the abattoirs (Table 3).

Purchasing cattle from an infected herd or a herd with unknown status increased the risk of detecting positive animals at slaughter (OR = 2.1 and 1.6 respectively, Table 3). This risk was further significantly increased for conventional farms when they purchased animals from herds with unknown status (OR = 4.1); however, this effect was not seen in organic herds. Conventional herds of small size had a non-significant reduced risk of being positive compared to small organic herds (OR = 0.84), but the risk was significantly lower in medium (OR = 0.48) and large (OR = 0.52) sized conventional herds as indicated by the interaction terms. Non-dairy herds showed an increased risk compared to dairy herds, but only if their herd size was medium (OR = 1.9).

### Evaluation of spatial predictions

The SatScan heat map (Figure 2a) showed that the observed spatial clustering was significant (P < 0.05). A subsequent visual comparison of the heat map for predicted probability (Figure 2b) with the observed status (Figure 2a) confirmed that the model prediction of *F. hepatica* infection matched the observed status (Figure 2a). This indicated that the model was able to reproduce the spatial patterns; and thus was able to capture the key parameters involved in the transmission of the disease. Both heat maps showed a high clustering in the Northern Jutland region, with slight deviations for the other regions.

## Discussion

This study was performed to estimate the annual proportion of cattle and herds that tested positive for *F. hepatica* infection at post-mortem meat inspection during the period 2011 to 2013. Additionally, risk factors were identified and quantified at herd level and the spatial variation *of F. hepatica* herd level infection in Denmark was explored further.

During the study period, per annum fasciolosis prevalence estimates at both animal and herd level increased suggesting that *F. hepatica* infection is a growing problem in Denmark as in other parts of Europe [20]. This rise in prevalence in recent years may be attributed to changes in farmers’ grazing strategies (e.g. use of more wetlands) or due to milder temperatures and wetter conditions, which affect transmission e.g. by increasing the size of the snail population as well as the period during which development may occur in the intermediate hosts [5,21]. Therefore, the grazing livestock are at risk of being exposed to higher levels of contaminated vegetation [5,22]. The prevalence of fasciolosis at animal level as estimated in this study is an underestimate of the true prevalence in the population due to the poor sensitivity of meat inspection [12]. Given the sensitivity of 63.2% as estimated in [12] and assuming a specificity of 100%, the true prevalence at animal level is 1.6 times higher than the measured prevalence.

Plotting of infected herds revealed high numbers of infected herds in North and Central Jutland region of Denmark. In prevalence studies, herd density is often explored as a potential risk factor, because herds situated closely to each other have an increased risk of between herd transmission [23,24]. Herd density can serve as a risk factor for transmission of *F. hepatica* infection, because the population of infected snails might expand and spread to the nearest farms. This may partly explain the high clustering of positive herds in the Central Jutland region, where herd density is high (>0.23 herds per kilometer^2^) [23]. However, spatial patterns also showed a large local clustering of infected herds in the Northern Jutland region with a lower herd density (<0.07 to 0.23 herds per kilometer^2^) [23]. This finding suggests that other parameters e.g. local environmental or meteorological factors could be driving the increasing herd prevalence as described in other studies [6,15].

Detection of *F. hepatica* infection varied between the abattoirs which is consistent with previous observations made on Danish abattoirs [13]. This variation may be due to differences in quality of liver inspection and line speed. Also, location of the abattoir in a high risk area for *F. hepatica* infection might play a role as most animals are slaughtered in a nearby abattoir.

Our study showed that both herd and environmental factors were associated with the presence of *F. hepatica* infection (Table 3), which is consistent with previous reports [15,25]. Buying cattle from positive herds was a significant risk factor for the presence of *F. hepatica* infection in slaughter animals (Table 3). Purchased infected animals, if left untreated, contaminate pastures during grazing and expose non-infected herd mates to *F. hepatica* infection when necessary environmental factors are present. The results also showed that in non-dairy herds the risk of being infected with *F. hepatica* was higher in medium sized herds (≥30 to < 100) and lower in larger sized herds (≥100) when compared to smaller sized cattle herds (<30). There is a significant positive association present between grazing and *F. hepatica* prevalence in cattle [26]. Hence, it is likely that the medium sized cattle herds in our study included heifer-raising herds and beef herds with frequent access to pastures which increased their risk to *F. hepatica* infection [26]. And the lower risk in large non-dairy herds may be because these herds included cattle from veal calf production with no access to grazing. However, this effect may also be due to some underlying management factors that were not measured in this study or because non-dairy herds slaughtered more animals compared to dairy herds. Organic herds were at significantly increased risk compared to conventional herds when they were of medium or large size. This might be explained by either more access to pasture or to lower treatment levels in organic herds.

Among environmental variables streams, wetlands and pastures were found to be positively associated with the presence of *F. hepatica* infection in Danish cattle (Table 3) which is also evident from other studies [15,27,28]. A Swiss risk factor study demonstrated that presence of streams and existence of snail habitats were positively correlated with occurrence of infected snails and fasciolosis on the farms [28]. Thus, the risk originates from cattle grazing on contaminated pastures nearby the streams [29]. The moist conditions of wetlands are known to favour the survival and spread of intermediate host snails, development of infection within the host snails and transmission of free living fluke stages [6,8]. A significantly positive association between *F. hepatica* infection in cattle and wetlands was shown in Brazil [30]. Streams, wetlands and pastures provide a perfect environment for the development and further expansion of host snail population, which subsequently increases the risk of *F. hepatica* infection in cattle [4,5,21]. The probability of being infected at dry-land areas was low (Table 3) likely due to absence of intermediate host snails and flukes. Additionally, a low risk of being infected when cropland was present on a farm has also been shown in another study and may be due to a lack of pastures, or less use of land for grazing, or generally drier farmlands [6,14]. In previous studies, presence of water-bodies and grassed areas has been classified as a risk factor for *F. hepatica* prevalence because these moist environments favour the intermediate host, *G. truncatula* [6,15]. However, in our study favourable environment conditions such as lakeshores, fresh-water meadows and grasslands showed no association with the presence of *F. hepatica* infection probably due to limited or no access to grazing in these areas. However, on the other hand, our result is consistent with finding that there is no association between the presence of forests and *F. hepatica* infection, which once again could probably be because of restricted grazing or due to absence of snails resulting from a lack of enough sunlight which limits the growth of food algae necessary for snails to breed [6,31].

The risk factor variables used in the model were able to predict the spatial patterns. However, the model showed deviations by predicting a higher probability of infection in a few areas, which was not seen in the observed prevalence data. This suggests that more work is required to evaluate and correctly capture the impact of the environmental and other local factors associated with the *F. hepatica* prevalence.

## Conclusions

The study showed an increase in annual herd level prevalence (2011–2013) indicating that *F. hepatica* infection is a growing problem in Denmark. Spatial analysis showed clustering of infected herds in some areas where herd density was not high suggesting that infection was possibly associated with local factors. Trade was a risk factor in medium-sized non-dairy herds and in herds from conventional systems that purchased cattle from infected farms. Presence of streams, wetlands and pastures on farms were significantly associated with the presence *F. hepatica* infection in cattle herds. Evaluation of the risk factor model showed that it reproduced spatial trends; however, the parameters included in the model did not fully capture the effect of environment on *F. hepatica* prevalence in Danish bovine herds.

## Abbreviations

CI: Confidence interval
DCD: Danish cattle database
IDW: Inverse distance weighted
OR: Odds ratio
P: Prevalence
RR: Relative risk
SE: Sensitivity
SP: Specificity
